# Estimation of the Spatial Suitability of Winter Tourism Destinations Based on Copula Functions

**DOI:** 10.3390/ijerph16020186

**Published:** 2019-01-10

**Authors:** Weiying Cai, Hui Di, Xingpeng Liu

**Affiliations:** 1 Northeast Asia Leisure Economy Research Center, The Tourism College of Changchun University, Changchun 130607, China; weiycai1982@163.com; 2 School of Environment, Northeast Normal University, Changchun 130024, China; dih717@nenu.edu.cn

**Keywords:** winter tourism, climate change, snow abundance index, meteorological suitability index, copula function, spatial suitability assessment

## Abstract

Climate and weather are important factors that determine winter tourism destinations and snow resources and temperature affect the income of the winter tourism industry. Against the background of climate change, abnormal fluctuations in climate elements bring a series of challenges for winter tourism and cause potential losses to the tourism industry. To effectively assess and plan winter tourism destinations, this study establishes the snow abundance and meteorological suitability indices from snow resource and weather conditions to express winter tourism resources, respectively. The coupling relationship of the two indices was used to analyze the spatial suitability of winter tourism destinations based on the copula function. By case analysis, it was found that the Frank copula one is the best fitting function for winter tourism suitability analysis. The Yushu–Jiutai–Yitong–Dongliao line is the boundary of spatial suitability in the study area. The eastern areas of the boundary have great potential for winter tourism and could strive to develop ice-snow projects, whereas the western regions are relatively weak. This study has guiding significance for winter tourism destination development and resource spatial layout.

## 1. Introduction

Climate and weather are important decision making factors for tourists and also influence the successful operation of tourism businesses, especially winter tourism [1,2,3,4]. In recent years, winter outdoor tourism activities have gradually gained popularity in China [5]. Both the government and enterprises have increased investment in winter tourism planning. The planning of winter tourism is affected by multiple factors (e.g., climate condition, terrain, land use, etc.); among them, climate and weather are the most important, and changes in these factors will directly affect tourism industry income. Winter tourism is strongly dependent on climate and weather. Under the background of climate change, changing factors lead to changing spatial suitability for winter tourism. To analyze the impact of weather and climate change on the industry, researchers usually develop a comprehensive index to measure the intensity of weather and climate. Weather and climatic index integrate two or multiple meteorological factors that represent weather and climatic characteristics. The weather index represents short-term weather characteristics, while the climate index represents medium and long-term climate characteristics. With the rapid growth of the economy and the increase in property density, the economic losses caused by extreme weather and climate events were increasing significantly. Several kinds of weather and climate indices were proposed by researchers and widely used in agricultural production, energy consumption, commodities, living consumption, health care, tourism, sports and leisure, transportation, insurance and finance and other fields [6,7,8].

Vast numbers of references have suggested that the China region is extremely sensitive to climate change [9]. Winter tourism is closely related to climate variations, especially in northeast China regions where resorts are heavily dependent on snow. Increasing temperatures and snow scarce winter seasons pose a big challenge for the winter tourism industry [10]. Increasing winter temperatures, lack of snow, decreasing snow cover and snow depth will result in a shorter skiing season [11], which could lead to smaller number of visitors and reduced revenues, and thus have severe economic impacts on winter tourism destinations [12]. Researches have conducted numerous studies on the impact of climate change on winter tourism. Gajićčapka and Srnec [13] analyzed the time analysis (fluctuations and trends) in different meteorological parameters related to snow (air temperature, total precipitation and air pressure) as well as snow parameters (snow cover frequency, duration and magnitude) themselves. Hoffmann et al. [14] proposed an econometric analysis model with linear regression and count data, which found a positive influence of the awareness of possible climate change effects on the scope of corporate adaptation. Damm et al. [10] analyzed the economic effects of technical snow production under future climate conditions. Dingeldey and Soboll [15] presented an interactive multi-agent scenario assessment model and used it to examine the future impact of climate change on winter tourism in the German and Austrian Alps. Gonseth [16] assessed the sensitivity of winter tourism consumption to changing snow conditions. Bonzanigo et al. [17] explored how to effectively integrate a climate change adaptation perspective with local discourses about sustainability and tourism, an increasing priority for policy-makers in the region and elsewhere. Besides, several studies analyzed the potential impact on snowmaking under climate change in the future [11,18,19,20]. The purposes of researchers want to accurately assess regional impact, vulnerability, risks and opportunities of climate change on winter tourism [21,22,23,24,25,26,27,28,29]. Therefore, the impact of climate change should be taken into account in winter tourism destinations planning.

To determine spatial suitability for winter tourism, it is very meaningful to analyze the spatial change in winter tourism resources under climate change. Snow resources and meteorological elements are the basic factors of winter tourism. With climate change, winter tourism in some regions of the world has been seriously affected by rising temperatures [2,30]. Climate change studies in Northeast China have proved that regional mean snow cover duration decreased at −2.7 days per decade and mean maximum snow depth decreased at −0.5 cm per decade, while low-temperature days (≤−25 °C) had a significant decreasing trend of −3.9 days per decade during 1961–2010 [31]. Rising temperature has led to snowfall reduction and a shorter winter tourism season, which has a certain negative impact on snow and ice projects in winter tourism destinations. It has shortened the development season and increased the costs of their tourism products. Therefore, analyzing the temporal and spatial changes in winter tourism resources and allocation can help decision-makers adjust their tourism management strategies.

Estimation of snow resources is one problem for winter tourism destinations. Snow resources include snow depth and snow cover days as two factors. Because of the significant signal difference between snow and other surface objects, snow cover is easily obtained by optical remote sensing such as MODIS data (e.g., MOD10C1). Compared with snow cover, snow depth data are difficult to obtain because of the poor penetration of optical remote sensing. Therefore, snow depth data are often traditionally obtained by weather station observations. With the development of passive microwave technology, passive microwave remote sensing data (e.g., Scanning Multichannel Microwave Radiometer (SMMR) and Special Sensor Microwave/Imager (SSM/I)) were widely applied to obtain snow depth due to their all-weather good penetrating ability, not being affected by cloud cover and high temporal resolution. The snow depth retrieved from passive microwave remote sensing data has been gradually promoted, and the brightness temperature gradient is the most representative algorithm [32]. Several studies have applied snow depth observation data to analyze the nonlinear relationship between snow depth and the brightness temperature gradient [33,34]. Che et al. [33] calculated the snow depth based on land use classification and the brightness temperature gradient and obtained the snow depth in China.

Besides snow resources, meteorological elements are equally important factors for winter tourism. Therefore, the weather index estimation for outdoor activities is another problem for winter tourism. In winter, outdoor snow sports are closely related to weather conditions, and among all the meteorological elements, air temperature and wind speed affect snow sports directly. Furthermore, snow quality is closely related to air temperature. High air temperature makes snow texture sticky and heavy, influencing sliding speed. Low temperature forms brittle ice that causes weak adhesion and easy skidding. For the influence of weather on outdoor sports, the present studies mainly focus on the degree of human body comfort, and the environmental comfort index was analyzed based on sensible human temperatures [35,36]. However, there were fewer studies on the weather conditions for human comfort in sports. In 2017, the Chinese Meteorological Administration issued a standard for the skiing meteorology index [37]. As one of the industries impacted by climate change, several studies analyzed the sensitivity of winter tourism to snow depth and temperature increase over the last decades [2,32,38,39]. The present researchers analyzed the impacts of climate change on winter skiing tourism areas and indicated that future climate change would shorten the skiing season and sharply reduce skiing visitors, causing economic loss in low altitude and low latitude skiable areas [40]. Several studies found that climate change has a negative impact on the sustainable development of winter tourism, skiing tourism and tourism vulnerability [17,41,42]. In the comprehensive evaluation of winter tourism resources, the present study mainly focuses on the qualitative analysis and characteristic description and lacks a precise quantitative analysis of spatial suitability assessment, which limited its application [43,44,45]. Under the background of climate change, winter tourism industries will face serious challenges. For government managers, how to rationally determine the winter tourism development zone under the influence of climate change has become an important problem that urgently needs to be solved. This has great significance for the sustainable development of the regional tourism industrial economy.

The winter tourism literature indicates that: (1) meteorological conditions and snow resources are two primary factors for winter tourism suitability assessment; however, very few studies have analyzed the temporal and spatial distribution coupling relationship of the two factors as well as the impacts on tourism planning. (2) The present research is mainly focused on the qualitative description of winter resources, lacking quantitative analysis of spatial suitability distribution, and is not suitable for tourism planning decisions. (3) The impact of climate change on winter tourism is uncertain. To avoid the impact of climate fluctuations on winter tourism, it is of great significance to obtain the spatially suitable development areas. To address these problems, this study proposes the meteorological suitability and snow abundance indices for winter tourism based on meteorological and snow data. Firstly, by analyzing the influence of weather conditions on snow cover and outdoor activities, air temperature, wind speed, relative humidity, and visibility are selected to establish the climate suitability index. Secondly, the depth and duration of snow cover are selected to characterize the spatial abundance of regional snow resources, which is used to establish a snow abundance index. To express the coupling effect of these two indices on winter tourism, the spatial joint probability of the two indices is calculated to express the spatial suitability for winter tourism using the Copula function. Finally, taking Jilin province as the area, this study analyzes temporal and spatial distribution and establishes the suitability of winter tourism resources. This study will provide a new method to analyze the impact of climate change on winter tourism. The proposed method and results will have a guiding significance for ice-snow tourism planning in winter tourism destination countries.

## 2. Materials and Methods

### 2.1. Study Area

**Figure 1:**
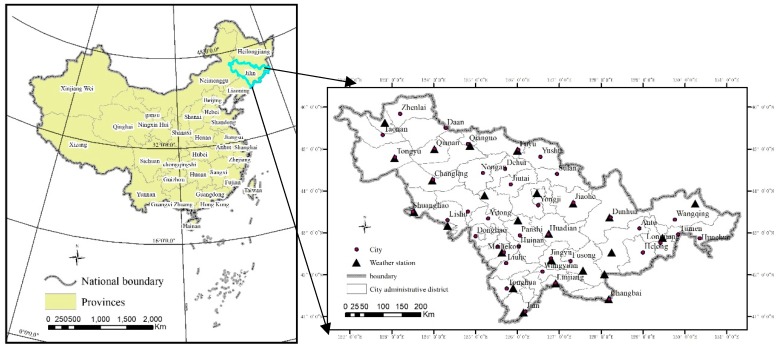
The location of the study area.

Northeast China is in a high latitude region affected by cold temperate and temperate continental monsoon climates. This area forms long cold winters and short warm summers. The winter tourism industry occupies the dominant financial position for this area. The study area is Jilin province located in the center of northeast China (40°52′–46°18′ N, 121°38′–131°19′ E) (Figure 1). The administration district area is 1.87 × 10^5^ km^2^. The winter in Jilin Province lasts half a year. It has significant potential for winter tourism because of abundant snow resources. Compared with other regions in northeast China, the meteorological conditions of Jilin Province are suited for outdoor activities, with good snow quality for snow sports. Therefore, Jilin Province is a major area for winter tourism, and winter tourism has gradually become a new growth pole to pull economic development into the province. During 2016 and 2017, Jilin province received 6.2 million tourists, and tourism income reached 116 billion CNY (Tourism Commission of Jilin Province data). To deeply plan and develop winter tourism resources, it is very necessary to estimate the spatial suitability for winter tourism in this area.

### 2.2. Methods

This study is mainly based on natural conditions (weather and snow) to assess the suitability of regional winter tourism development. To analyze the meteorological conditions and snow resources of Jilin province, the data sources include remote sensing images and local meteorological records. The snow depth data were extracted from the long-term snow depth dataset of China [46]. The original snow data sources are passive microwave images since 1980 obtained from the US National Snow and Ice Data Center (NSIDC). The original long time series dataset of China snow is (1) passive microwave remote sensing SMMR (1980–1987), (2) SSM/I (1987–2007) and (3) SSMI/S (2008–2014). Meteorological data include daily air temperature and wind speed (2 m height) obtained from the Chinese Meteorological Administration (daily data from 1971 to 2016).

#### 2.2.1. Meteorological Suitability Index (MSI)

Air temperature and wind speed are two important factors that directly influence snow quality and outdoor snow sports in winter. When the mean air temperature is about −12 °C, and air relative humidity less than 80%, it will form power snow which is suitable for skiing. The hardness and softness of snow are closely related to air temperature. When the temperature rises, the snow surface will melt gradually under the action of sunlight and the continuous rolling of skis, which cause snow becomes soft; while the extreme cold weather will lead to water condensation and even a thicker ice crystalline layer in the snow, which cause snow to become hard. The present study indicates that when the air temperature is too low (≤−20 °C) tourists feel uncomfortable [37,47,48], and when it is too high (≥2 °C), it influences snow quality and snow cover days [47,49]. Outdoor snow sports are directly restricted by high wind speed. When wind speed reaches a certain level, outdoor activities are limited. When there is wind, the body temperature will be lower. Therefore, when the minimum temperature reaches −16 °C, visitors feel significantly uncomfortable. When the wind-force is less than scale 2 to 3, the skiing is suitable, and when the wind-force is more than scale 5, the skiing will be dangerous. Air relative humidity and visibility also limit outdoor activities [37]. The influence of weather on the spatial and temporal distribution of winter tourism is analyzed by the coupled analysis of air temperature, and wind speed in this study. The classification of a single factor is referred to the meteorological skiing index issued by the Chinese Meteorological Administration [37]. In this study, the fuzzy inference and following steps are used to analyze MSI.

Step 1: Determining the evaluation factors and their grades.

Four factors daily maximum temperature (T), wind speed (W), air relative humidity (H) and visibility (V) were selected to describe meteorological suitability. The factors and their grades are shown in Table 1.

Step 2: Constructing the fuzzy membership function of factors

The Gaussian membership function, Z-shaped, and S-shaped membership function were used to establish the fuzzy membership of factors. The symmetric Gaussian function depends on two parameters σ and *c* as given by:

(1) $$f\left(x,\sigma ,c\right)=e^{\frac{-{\left(x-c\right)}^{2}}{2\sigma^{2}}}$$

Z-shaped and S-shaped membership are spline-based functions. The parameters a and b locate the extremes of the sloped portion of the curve as given by:

(2) $$g\left(x,a,b\right)=\{\begin{array}{c} \begin{array}{cc} 1 & x\leq a\\ 1-2{\left(\frac{x-a}{b-a}\right)}^{2} & a\leq x\leq \frac{a+b}{2}\\ 2{\left(\frac{x-b}{b-a}\right)}^{2} & \frac{a+b}{2}\leq x\leq b \end{array}\\ \begin{array}{cc} 0 & x\geq b \end{array} \end{array}$$

(3) $$h\left(x,a,b\right)=\left\{ \begin{array}{cc} 0 & x\leq a\\ 2{\left(\frac{x-a}{b-a}\right)}^{2} & a\leq x\leq \frac{a+b}{2}\\ 1-2{\left(\frac{x-b}{b-a}\right)}^{2} & \frac{a+b}{2}\leq x\leq b\\ 1 & x\geq b \end{array}\right.$$

The parameters σ, *c*, a and b will be established based on the boundary value in Table 1.

Step 3: Determining the weights of factors

Previous studies have shown that air temperature, wind speed, and visibility are all restrictive factors of outdoor activities, and they play an equally important role in winter tourism [37]. The air relative humidity is a non-restrictive factor of winter tourism. Therefore, the weight of this study is assigned as

A = [0.3, 0.3, 0.3, 0.1]

Step 4: By using the membership formula, the daily fuzzy membership of each meteorological factor of MSI at different levels can be obtained using Equation (4):

(4) B=A°R

where ° is fuzzy operator symbols, A is a row matrix of factor weights, R is a matrix of fuzzy sets, which is calculated using Table 2. The MSI is divided into three levels and assigned value 1, 2 and 3. The values 1, 2 and 3 mean high-suitability, medium-suitability, and low-suitability, respectively. The daily MSI is determined according to the principle of maximum membership degree (Equation (5)). To facilitate the calculation of MSI, this study compiled the MSI using MATLAB 2016b (MathWorks, Natick, MA, USA).

(5) MSI= max (B)

#### 2.2.2. Snow Abundance Index (SAI)

The abundance of snow resources in a region is mainly expressed by snow thickness and duration. Therefore, daily snow depth and duration are used to calculate the snow abundance index. Winter tourism depends on the amount of snow, only when the snow reaches a certain depth and lasts for a period of time can tourists be attracted. Experience and researchers have found that only when the snow depth reaches more than 10 cm, can greatly reduce the cost of artificial snowfall in the ski resort and get economic benefits [40,50]. It is generally considered that snow sports need at least 30 cm of snow depth [40]. Witmer [51] suggested that a skiable area with at least 30 cm snow depth and 100 days duration per year would be suitable for the skiing industry. If the snowfall is reduced in one year, scenic areas can make snow artificially to satisfy tourists. The snowfall determines the snowmaking cost and tourism income. Therefore, the winter tourism industry needs to obtain the spatial distribution of snow resources. At present, several studies have obtained large-scale spatial snow resources based on remote sensing images and proved their feasibility [32,33,34]. Based on the snow data obtained from local remote sensing images, the snow abundance index was designed by coupling daily snow depth and duration in this study (Equation (6)). Several studies have provided a method to classify snow depth [52]. In this study, based on previous researches [50,51,52] and according to the actual situation of snow depth in Jilin Province, the snow depths were divided into <10, 10–20 and >20 three classes:

(6) $$MSI=\{\begin{array}{c} \begin{array}{cc} 1 & SD\geq 20\;AND\;SC\geq 60\; \end{array}\\ \begin{array}{cc} 2 & SD\geq 10\;AND\;SC\geq 30\; \end{array}\\ \begin{array}{cc} 3\;\;\;\;\;\;\;\;\;\;\;\;\;\;\;\;\;\;\;\;\;\;\;\;\;\;\;\;\;\;\;\; & others \end{array} \end{array}$$

where *SD* is the snow depth (cm) and *SC* is the snow cover days (d). The values 1, 2 and 3 mean high-abundance, medium-abundance, and low-abundance, respectively.

#### 2.2.3. Copula Function

Copula functions describe nonlinear relationships among multivariate data and model sample nonlinearly interrelated multivariate data [53]. They are functions that couple the joint distributions to their marginal distributions. Recently, copula methods have been extensively applied for finance, natural sciences and engineering, etc. To analyze the combination of MSI and SAI in space, the joint distribution of two indices is calculated with Copula functions to obtain the suitability degree for winter tourism regions.

Copulas provide a method for measuring the dependence between variables. Sklar [54] described the function relationship between a Copula C and a cumulative distribution function. Suppose there is a bivariate domain, and $F_{X}\left(x\right)=P\left[X\leq x\right]$ and $F_{Y}\left(y\right)=P\left[Y\leq y\right]$ are cumulative distribution functions of the random variables X and Y. Their joint distribution is $F_{XY}\left(x,y\right)=P\left[X\leq x,Y\leq y\right]$. Then, on the basis of mathematical theory, there is a unique function C,$\;F_{XY}\left(x,y\right)=C\left(F_{X}\left(x\right),F_{Y}\left(y\right)\right)$, and C is called a copula function. Thus, if function C was deduced, the joint distribution F(x,y) of variables can be derived from their marginal distributions, $F_{X}\left(x\right)$ and $F_{Y}\left(y\right)$. Furthermore, let the probabilities $u=F_{X}\left(x\right)$ and $v=F_{Y}\left(y\right)$; we can take $x=F_{X}^{-1}\left(u\right)$ and $y=F_{Y}^{-1}\left(v\right)$. Then, $\;F_{XY}\left(x,y\right)=F\left(F_{X}^{-1}\left(u\right),and\;F_{Y}^{-1}\left(v\right)\right)=C\left(u,v\right)$ is a copula (*Sklar’s theorem*) if a two-dimensional function, C:[0,1]×[0,1]→[0,1], meets three conditions:

(1) C(0,v)=C(u,0)=0;
(2) C(1,v)=C(u,1)=1;
(3) For all $0\leq u_{1}\leq u_{2}\leq 1$ and $0\leq v_{1}\leq v_{2}\leq 1$, $C(u_{2},v_{2})-C(u_{2},v_{1})-C)u_{1},v_{2})+C(u_{1},v_{1})\geq 0$.

There are two categories of widely used copulas: ellipse and Archimedean [55]. Ellipse copulas, such as the Gauss- and *t*-copulas, can be produced by known multivariate distributions. Archimedean copulas are produced from different generators (φ) based on the definition of the Archimedean copula [56]. At present, Archimedean copulas are widely used in actual applications because they can model dependence in arbitrarily high dimensions with only one parameter, governing the strength of dependence. The one-parametric Archimedean Copula is expressed as:

(7) $$C\left(u,v;\theta \right)=\varphi^{-1}\left[\varphi \left(u,\theta \right)+\varphi \left(v,\theta \right);\theta \right]$$

where C is a Copula function, φ is the generator function, and $\varphi^{-1}$ is its pseudoinverse. φ:[0,1]×Θ→[0,∞), φ(1)=0. θ is a generator function parameter within parameter space Θ. Further important Copula features and the theoretical background can be found in Nelsen [57], who provides a detailed introduction to Copula functions.

Kendall’s τ is the rank correlation coefficient. It can be calculated from the available observation samples as:

(8) $$\tau ={\left(\begin{array}{c} N\\ 2 \end{array}\right)}^{-1}\overset{N}{\underset{j=1}{\sum }}\overset{j}{\underset{i=1}{\sum }}sign\left[\left(x_{i}-x_{j}\right)(y_{i}-y_{j})\right]$$

where sign = 0 if $\left[\left(x_{i}-x_{j}\right)(y_{i}-y_{j}\right)]=0$, sign = 1 if $\left[\left(x_{i}-x_{j}\right)(y_{i}-y_{j}\right)]>0$, and sign = −1 if $\left[\left(x_{i}-x_{j}\right)(y_{i}-y_{j}\right)]<0$ and i,j=1,2,⋯,N [58]. The corresponding Copula function coefficient θ can be calculated using the functions in the right column of Table 3.

In this study, MSI and SAI are used to describe the suitability degree of winter tourism. The joint probability of the two indices in the space is analyzed based on Copula functions. The root mean square errors (RMSEs) are used to identify the most appropriate Copula function, which is calculated from the theoretical and the empirical joint non-exceedance probabilities [59]. The RMSEs are determined using all of the observed samples and empirical non-exceedance probabilities of events:

(9) $$RMSE=\sqrt{\frac{1}{N}\overset{N}{\underset{i=1}{\sum }}{\left[P_{c}\left(i\right)-P_{o}\left(i\right)\right]}^{2}}$$

where $P_{c}\left(i\right)$ is the joint probability of the *i*^th^ joint observation value calculated by the Copula function, N is the observation samples, and $P_{o}\left(i\right)$ is the empirical joint nonexceedance probability, calculated as follows [58,60].

(10) $$F_{XY}\left(x_{i},y_{i}\right)=P\left(X\leq x_{i},Y\leq y_{i}\right)=\frac{\sum_{m=1}^{i}\sum_{l=1}^{i}N_{mj}-0.44}{N+0.12}$$

where $N_{mj}$ represents the number of occurrences of $\left(x_{m},y_{j}\right)$ with $x_{m}<x_{i}$ and $y_{j}<y_{i}$, *i* = 1, …, *N* and m,j∈[1,i]. *N* is the sample size. In the calculation process, the pairs $\left(x_{m},y_{j}\right)$ are arranged in ascending order with respect to $x_{m}.$

## 3. Results

### 3.1. Changing Temperature and Snowfall under Climate Change

In this study, the winter snowfall, air temperature and wind data are used to assess the suitability of winter tourism in Jilin Province. The snowfall data were mainly used to analyze the influence of snow depth on winter sports, and the air temperature and wind determine the comfort level of outdoor sports. Several research studies show that snowfall and air temperature have changed significantly in Northeast China with climate change [31,61]. As part of Northeast China, the spatial distribution of snow, air temperature, and wind in Jilin province has also changed significantly. Revealing these changes can provide important informational support for winter tourism management in Northeast China. Therefore, in this study, the spatial and temporal distribution of snow, air temperature, and wind in Jilin province were analyzed using meteorological data from 1971 to 2016 (from December to February of the next year).

**Figure 2:**
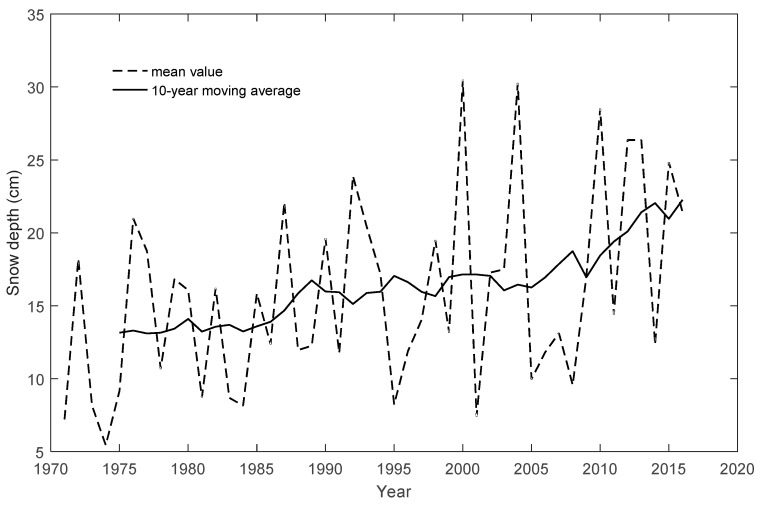
Snow depth change from 1971 to 2016 in Jilin Province.

**Figure 3:**
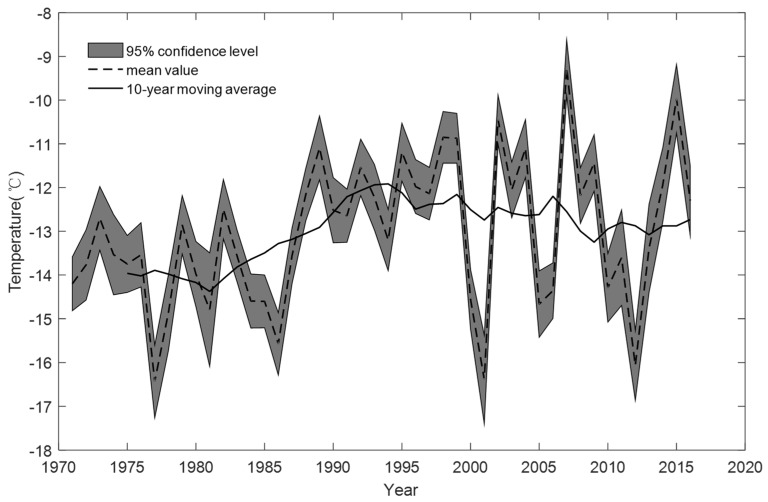
The temperature change from 1971 to 2016 in Jilin Province.

Figure 2 shows the snowfall depth in Jilin Province varying from 5 to 30 cm, and the snowfall in winter increased by 1.3 mm/10a. Figure 3 shows that the temperature in Jilin Province varied from −16.4 °C to −9.2 °C, and the average temperature increased 0.27 °C/10a in the study area, including 0.25 °C/10a in the west, 0.26 °C/10a in the middle and 0.29 °C/10a in the east. The overall air temperature and snowfall change is beneficial to winter tourism. However, the annual temperature and annual snowfall fluctuated strongly. This will not be a disadvantage to winter tourism.

Climate change has favorable and unfavorable conditions for winter tourism. To avoid the influence of unfavorable factors, we need to distinguish the regions that are more suitable for development of winter tourism space. Therefore, the two indices, namely, MSI and SAI, were used to analyze the suitability of winter tourism space.

**Figure 4:**
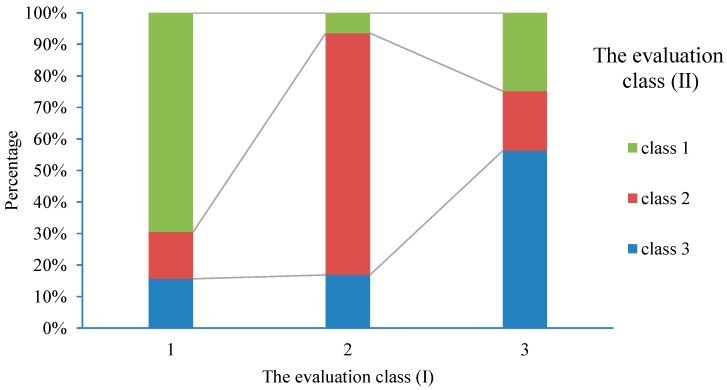
The cross validation of evaluation results in class (I) and class (II).

MSI evaluated comprehensively the suitability of meteorological environment using fuzzy inference. It is the weather index for suitable winter tourism. By coupling analysis of daily maximum air temperature, mean wind speed, air relative humidity, and visibility in winter, the spatial distribution of annual MSI was obtained in the study area from 1971 to 2016. The evaluation results (class (I)) were verified using the method provided by the literature [38] (class (II)) (Figure 4).

Figure 4 shows that the percentage difference between the two evaluation methods was 27.8%, 37.5% and 37.3% in the three grades of high (value1), medium (value1) and low (value3), respectively. Overall, 65.8% of the evaluation results are coincident. The major reasons are that visibility and relative humidity are considered in this study. Therefore, the degree of suitability predicted in this study is lower than that provided with the literature [38]. But in terms of temperature and wind speed, the evaluation results of the two methods are consistent.

By analyzing the mean value of annual MSI in the space, it is found that the maximum frequencies of high-suitability and medium-suitability degrees in the study area are 27.1% and 26.1%, respectively. The high frequency of high-suitability areas is mainly distributed in the eastern region and gradually decreases to the west, whereas the medium-suitability area is mainly distributed in the central region and gradually decreases to the east and west. It can avoid the inherent subjectivity of objective selection based on experience and make evaluation more scientific and reasonable. Compared with the analytic hierarchy process and risk matrix method, MSI calculated by fuzzy inference is more objective, and the evaluation results are closer to the actual situation.

**Figure 5:**
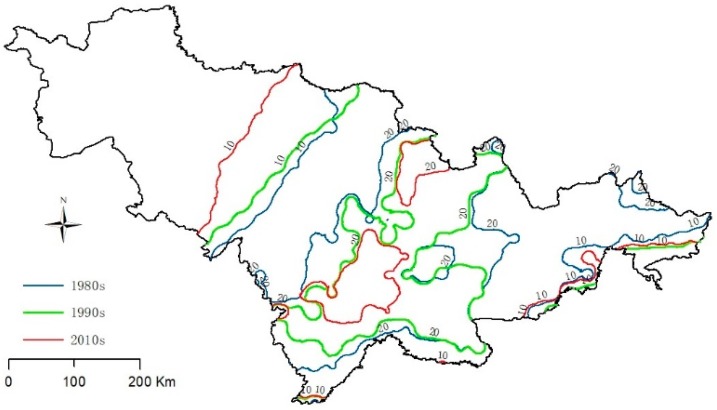
The contour of 10 and 20 cm snow depths in the 1980s, 1990s and 2010s.

The SAI indicates the snow richness for outdoor activities in winter. It found an obvious spatial distribution difference between snow depth and duration in eastern, central and western Jilin province. The snow cover in the eastern region lasted for a long time, followed by the central plain area. The western region has the shortest snow cover time. The snow depth and snow cover decrease from southeast to northwest. The snow resource is mainly concentrated in the central-eastern study area. The maximum durations of snow depth exceeding 10 cm and 20 cm were reached at 35 and 79 days, respectively. In the central part of Jilin Province, the 10 cm snow depth lasted for 3–10 days, and the 20 cm snow depth lasted for 10–30 days. In the western part of Jilin, the duration of 10 cm snow is 1–5 days, and 20 cm snow depth is 5–10 days. By analysis of the snow depth contour exceeding 10 and 20 cm (Figure 5), it was found that the areas of 10 cm snow depth increased in the 1980s, 1990s, and 2010s, but the areas of 20 cm snow depth decreased in the same periods. This means that the number of heavy snow days is decreasing and that the number of light snow days increased in these periods.

### 3.2. The SAI and SMI Joint Probability

**Figure 6:**
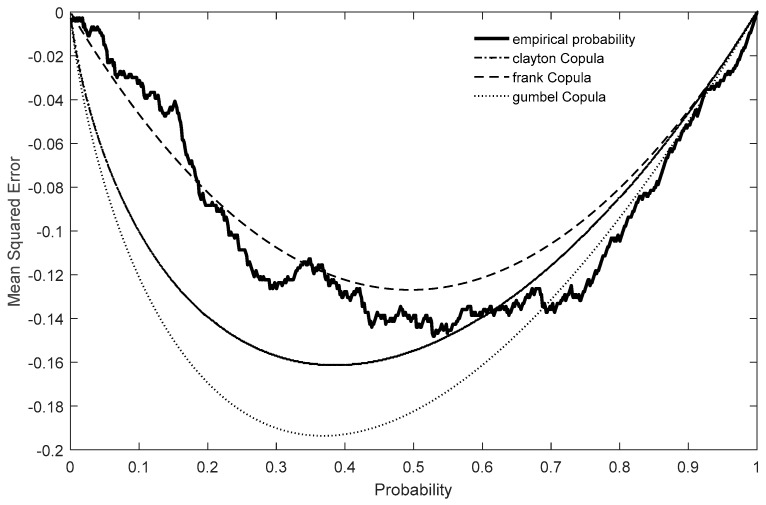
The verification results of copula functions.

To obtain stable income, a suitable area for winter tourism development should be located where the meteorological factors steadily change annually and the winter tourism resources are suitable for development. Therefore, the coupling of annual MSI and SAI from 1971 to 2014 was used to analyze the suitability of winter tourism. To analyze the spatial coupling relationship between the annual SAI and SMI, the joint probability of the two indices was calculated based on Copula functions, and then the winter tourism suitability degree was established. In this study, the annual SAI and SMI were used to establish the copula function. The RMSEs determined by all of the observed samples identified the most appropriate copula function. By comparing the copula functions in Table 1, the results show that the RMSEs are 0.298 (Clayton), 0.241 (Frank), and 26.264 (Gumbel) at the 0.05 significance level (Figure 6). Therefore, the Frank copula function was selected to calculate the joint probability, and *θ* = −1.471 was calculated using the study area sample data. Through analysis, it is found that the high-suitability region is located in central and eastern Jilin Province (the joint probability >60%), the low-suitability region is located in west Jilin province (the joint probability <40%), and Yushu–Jiutai–Yitong–Dongliao is the boundary line. In central and eastern Jilin Province, the spatial distribution matching degree of the annual MSI and SAI is high and winter tourism resources are abundant (Figure 6). According to the two indices, the western region is weak for developing winter tourism because of low air temperature and the lack of snow resources.

**Figure 7:**
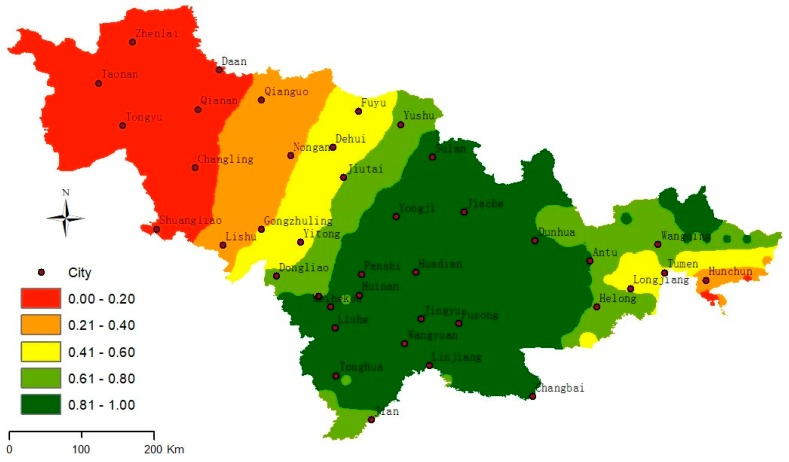
The joint probability spatial distribution of the SAI and MSI in the study area. The suitability degrees were classified as 0.00–0.20 (very low), 0.21–0.40 (low), 0.41–0.60 (medium), 0.61–0.80 (medium-high), and 0.81–1.00 (high).

Snow-based winter tourism has long been dealing with variability in natural snowfall and seasonal temperatures, which has led to early adaptive interventions and investments [62]. Figure 7 shows that winter tourism suitability in western Jilin is low, mainly due to lower air temperature in the winter (annual air temperature below −13 °C, and annual rainfall below 10 cm), so it is not suitable for development of ice and snow tourism. Due to current limitations on temperatures for snowmaking (usually below −2 °C) [63], western Jilin Province is suitable for artificial snowmaking. However, due to the lack of regional competitiveness, the attractiveness of its scenic spots is not as attractive as that of the central and eastern region. The most suitable areas for development of ice and snow tourism are the central and eastern regions of Jilin Province, mainly because they are rich in snow resources (snow depth is over 15 cm) and annual air temperature (over −10.4 °C) suitable for outdoor activities and long snow retention time (over 150 days), which makes for less restrictions on the development of tourism and for higher winter tourism income. This is the most advantageous area for the development of the ice and snow industry. There are 38 large ski resorts in Jilin province, and all are located in this area. Jilin province has designated 27 key winter tourist attractions, 94% of which are located in the region. These analysis results are consistent with the actual Jilin Province situation.

Copula functions characterize the spatial interdependence of two factors. It could analyze the joint recurrence probability of the two indices in space in this study. The higher the joint recurrence probability is, the better the spatial suitability of winter tourism is, which means that this area was less affected by climate change, and it is suitable for the development of winter tourism. Compared with the traditional comprehensive evaluation method (e.g., weighted overlay), the parameters of this method are obtained by calculating historical data, which can avoid the shortcomings of subjectivity in the weighted overlay.

## 4. Discussion

To get the spatial distribution of snow resources for winter tourism assessment, remote sensing data were used to calculate snow depth. Snowfall data from meteorological stations were used to verify the accuracy of snow depth obtained from remote sensing data in space. The spatial location of winter tourism scenic spots and skiing areas in Jilin province verifies the assessment results.

### 4.1. Spatial Snow DEPTH verification

**Figure 8:**
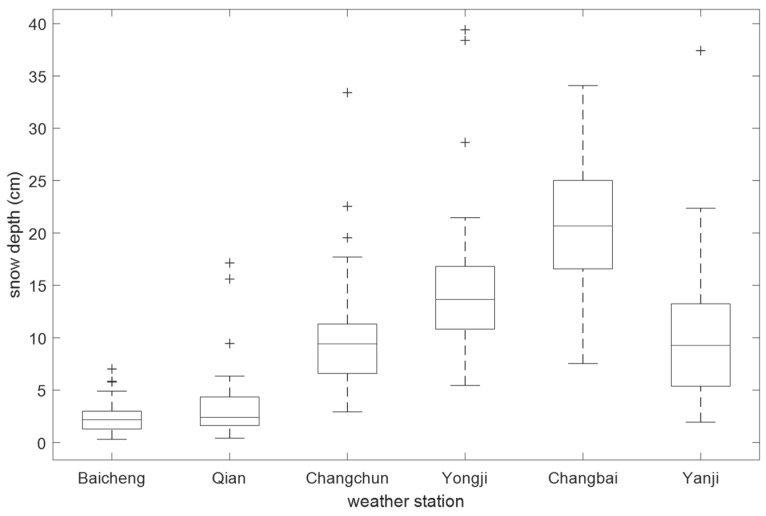
The snowfall from weather stations in the study area.

According to the data from meteorological stations, snowfall is increasing gradually from west to east in Jilin province from the boxplot (Figure 8). 

The trend obtained from two data sources is identical in the study area. From the variety of snowfall, there is little difference in depth in western Jilin Province, but the snow depth varies greatly in eastern Jilin Province. The reasons are that the western Jilin Province belongs to the arid area, the annual snowfall in this area are 29.3–40 mm in the winter, while this area belongs to a plain area with little snow cover. For the eastern region, this area belongs to a humid area with diverse land use and complex topography, which forms several local microclimates. This area has the abundant precipitation (100–190 mm) in the winter. With the monsoon and the topography, this caused a great change in the winter snow depth.

**Figure 9:**
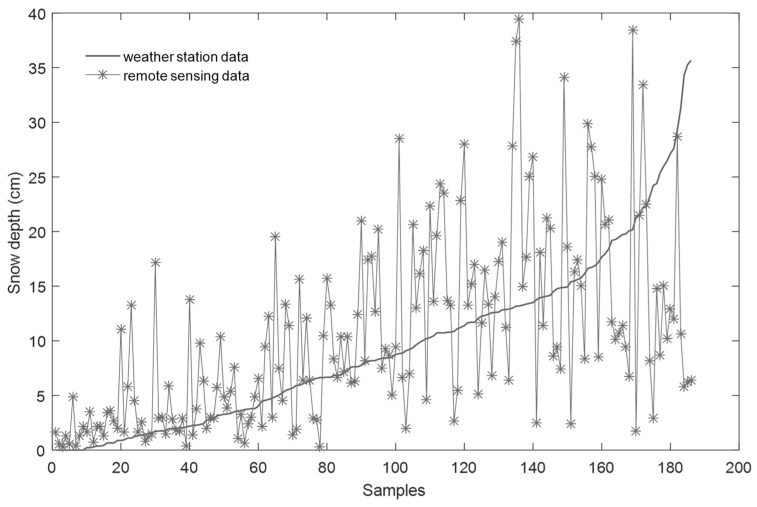
Comparing the two sources of snow resources data.

A total of 186 snowfall records from 1980 to 2014 in six meteorological stations were selected to verify the snow depth results. Based on the ascending observational data of meteorological stations, the tendencies of snow depth in the two sources are consistent in spatial distribution (Figure 9), but there is a certain difference. This is because meteorological observation data mainly record one snowfall, and the snow depth from remote sensing data may be multiple-snowfalls in the same space. Therefore, there will be a certain gap between the two data sources.

### 4.2. Winter Tourism Verification

**Figure 10:**
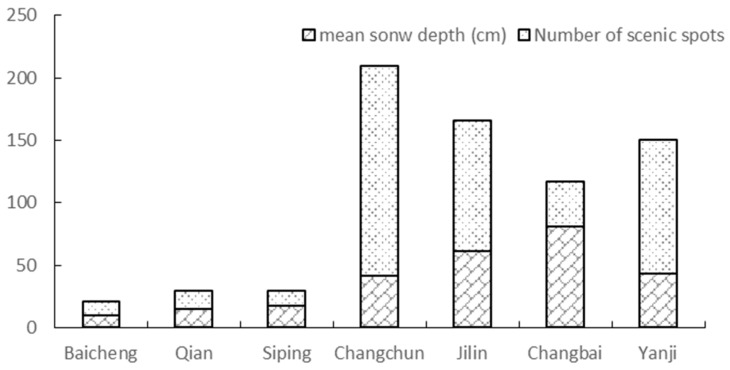
The number of winter tourism scenic spots and skiing areas in Jilin province.

To verify the reliability of winter tourism resources, this study obtained the spatial location of winter tourism scenic spots and skiing areas in Jilin Province (Figure 10). Their spatial distribution shows that these spots are mainly concentrated on the snow abundant areas. In addition to Changchun, the winter tourist attractions in the eastern region are obviously more than in the west. This shows a decreasing trend from east to west consistent with the spatial distribution suitability degree obtained in this study. Changchun is provincial capital, which located in the central area of Jilin Province. The snow resources in this area are at a medium level. However, due to its developed economy and good tourism infrastructure, it has great attraction for winter tourists. The development of scenic spots on other areas is mainly based on the exploitation of ice and snow resources.

By comparing these locations with the classification results of this study, Cohen’s kappa coefficient was used to measure the results (Equation (11)):

(11) $$k=\frac{p_{0}-p_{c}}{1-p_{c}}$$

where k is the kappa coefficient, $p_{0}$ is the observed agreement among raters, and $p_{c}$ is the hypothetical probability of chance agreement. By calculating the consistency and coefficient of the actual suitability of winter tourism scenic spots and skiing areas using Equation (7), it is found that k = 0.771 > 0.70, which means that the assessment results are highly consistent with the actual situation.

## 5. Conclusions

To analyze the spatial suitability distribution of winter tourism, the daily maximum temperature, mean wind speed, relative humidity, and visibility are used to establish the MSI, and snow depth and duration are used to establish the SAI. The coupling relationship of the two indices is used to analyze the suitability degree of winter tourism based on a copula function, and then the distribution of winter tourism resources can be obtained and used in tourism planning.

According to the MSI analysis, the high-suitability area mainly distributed in the eastern region, whereas the medium-suitability area mainly distributed in the central region. The SAI analysis shows that the snow resource mainly concentrates on central-eastern Jilin province, and the snow resources in the western region are less. Through the analysis of snow and weather data from 1980 to 2014, it is found that the *Frank* is the suitable function to analyze the coupling relationship between MSI and SAI with an error of 0.241 at the 0.05 significance level. From the MSI and SAI coupling, it is found that central-eastern Jilin province is suitable for developing tourism with a tendency to decrease from the southeast to the northwest. Yushu–Jiutai–Yitong–Dongliao is the main boundary line of winter tourism resources. From the annual variations of MSI and SAI, there is high suitability in weather conditions and snow resources in the eastern region, which is suitable for the development of winter tourism. On the contrary, it has a few snow resources and large inter-annual fluctuation in the western region, therefore, the construction of long-term ski resort in this area will be very expensive. The low temperature in the west makes it possible to develop the ice-related tourism industry. That is, the eastern area is suitable for developing winter tourism, and the suitability degree of the western region is weak from little snowfall and strong wind.

Previous studies have shown that climate change will bring positive and negative impacts on winter tourism in different regions. There are also large differences in seasons. Climate change causes a lot of losses to the tourism industry in some regions while improving the suitability of tourism in other regions [64]. Combined with the change of 10 cm snow depth contour map (Figure 5) and the average temperature in winter in Jilin Province (Figure 3), it can conclude that the mean temperature in winter in Jilin Province showed an increasing trend (0.29 °C/10a). In terms of snow depth, winter precipitation in Jilin Province showed an increasing trend, and the 10 cm snow depth contour has been moving westward from the 1980s to 2010s. Therefore, combined with these two trends, it can be predicted that the winter tourism suitability in the central -western regions of Jilin Province will increase under the influence of climate change. These conclusions are consistent with the previous research results [65]. Therefore, the suitability boundary of winter tourism will move westward.

In this study, MSI can be used to guide tourists to travel outdoor activities in winter. SAI can be used for regional snow resources assessment and water resources assessment. This study proposed a spatial analysis method for winter tourism suitability that can be used for winter tourism planning. The study results revealed the spatial constraints of meteorological and snow resources on winter tourism. This study will have a potential impact on the attractiveness analysis of winter tourist destinations and the development of winter tourism industry. This study could provide decision support for the tourist choosing winter tourism destinations, the construction of winter ski resorts and the spatial layout of the winter tourism industry. In a further study, the winter tourism spatial suitability and its impact on the industrial economy will be studied by taking into account land use, transport, terrain, and the socioeconomy.

## Figures and Tables

**Table 1:** The factors and their grades in MSI.

Factors	High	Middle	Low
H(°C)	[−12, −8]	[−16, −12) OR (−8, 2]	>2 OR < −16
W(m/s)	<5.4	[5.4, 10.7]	>5
H(%)	[50, 60]	[30, 50) OR (60, 80]	>80 OR < 30
V(km)	>1	[0.5, 1]	<0.5

**Table 2:** The membership functions for factors.

Factors	High	Middle	Low
H(°C)	f(x, 2,−10)	max(f(x,2,−14),f(x,2,−3))	max(h(x,−2, 2),g(x,−18,−12))
W(m/s)	g(x,3, 5)	f(x,2, 7.5)	h(x,7, 10)
H(%)	f(x,5,55)	max(f(x,10, 40),f(x,10,70))	max(h(x,75, 85),g(x,25,35))
V(km)	h(x,0.5, 1)	f(x,0.1, 0.5)	g(x,0.1,0.5)

**Table 3:** Archimedean Copula functions, their generators, and connections to Kendall’s.τ considered for this study

Kinds	Copula Function (C)	Generator (φ(t))	Range θ∈	Kendall’s τ
Frank	$\frac{-1}{\theta }ln\left[1+\frac{\left(e^{-\theta u}-1\right)\left(e^{-\theta v}-1\right)}{e^{-\theta }-1}\right]$	$\ln\left(\frac{e^{-\theta t}-1}{e^{-\theta }-1}\right)$	(−∞,+∞)\{0}	$r=1-\frac{4}{\theta }\left[1-\frac{1}{\theta }\int_{0}^{\theta }\frac{t}{e^{t}-1}dt\right]$
Clayton	${\left[u^{-\theta }+v^{-\theta }-1\right]}^{\frac{-1}{\theta }}$	$t^{-\theta }-1$	[0,∞)	$\tau =\frac{\theta }{\theta +2}$
Gumbel	$exp\left\{-{\left[{\left(-lnu\right)}^{\theta }+{\left(-lnv\right)}^{\theta }\right]}^{\frac{1}{\theta }}\right\}$	${\left(-lnt\right)}^{\theta }$	[1,∞)	$\tau =1-\theta^{-1}$
